# What are the key drivers to promote entrepreneurial intention of vocational college students? An empirical study based on structural equation modeling

**DOI:** 10.3389/fpsyg.2022.1021969

**Published:** 2022-10-28

**Authors:** Xinchen Niu, Zhining Niu, Mengmeng Wang, Xueshi Wu

**Affiliations:** ^1^School of Education, Jiangxi Science and Technology Normal University, Nanchang, China; ^2^School of Economics and Management, Weifang University of Science and Technology, Shouguang, China

**Keywords:** creativity, entrepreneurial intention, entrepreneurial attitude, social support, risk propensity

## Abstract

In order to alleviate the increasing employment pressure of vocational college students, the current study is an attempt to explore the factors of entrepreneurial intention affecting vocational college students. The study investigates whether entrepreneurial self-efficacy and attitude mediate this relationship between creativity and entrepreneurial intentions using the theory of planned behavior (TPB). In particular, this research also examines whether risk propensity moderates the relationship. An empirical survey is conducted and a total of 500 valid questionnaires are collected through online platforms. The data is analyzed by employing Partial Least Squares Structural Equation Modeling and SPSS20.0. The results indicate that self-efficacy is the strongest antecedent of entrepreneurial intention. Social support is found to directly influence entrepreneurial intention significantly, while the direct effect of creativity on intention is very marginal. Moreover, the results of the mediation analysis show that the relationship between creativity and entrepreneurial intention is fully mediated by self-efficacy and attitude, while the effect of social support on entrepreneurial intention is partially mediated. Specifically, the moderation effect of risk propensity on the relationship between creativity and entrepreneurial intention is acknowledged. Concrete suggestions are proposed for vocational colleges and governments to promote students’ entrepreneurial intentions. Finally, implications for the findings are provided.

## Introduction

According to the statistics of the Ministry of Education of China, there will be 10.76 million fresh graduates from colleges and universities at all levels in 2022 in China, a net increase of 1.67 million over 2021(Ministry of Education of People's Republic of China, 2022). This is the first time that the number of college graduates has exceeded 10 million, and also the year with the largest increase in recent years. The sharp increase in the number of employees has aggravated the pressure on employment. Meanwhile, the global spread of the epidemic has made the employment situation increasingly severe (Akter et al., 2020; Guerrero et al., 2020). Accordingly, it has great reference significance for studying the entrepreneurial intentions of other countries due to the large number of Chinese college graduates.

Entrepreneurship is a competitive behavior that drives new markets, employment creation, and innovation. From the perspective of individuals, entrepreneurship can alleviate the employment pressure of vocational college students and cultivate an innovative spirit. From the perspective of society, entrepreneurship is the driving force of regional economic sustainable development (Neneh, 2020; Bui Ngoc Tuan and Pham, 2022; Muammar and Maker, 2022). In higher education, entrepreneurial groups come from two categories: students from general higher education and students from higher vocational education. Compared with students from general higher education, students from higher vocational education are more inclined to choose to start a business after graduation (Mahmood et al., 2018; Phraudomsitthinayok, 2019). The main reasons are as follows: Firstly, the students cultivated by vocational colleges meet the needs of technical talents due to industrial upgrading and economic structural adjustment. The application-oriented is more prominent, and the practical operation ability is stronger than that of undergraduate students (Younis et al., 2020). Secondly, vocational college students have specific vocational skills as well as a solid knowledge foundation and entrepreneurial potential. They are more willing to change their situation compared with highly educated people, which often urges them to consider entrepreneurship.

Although governments at all levels have issued a series of preferential policies to encourage students to participate in entrepreneurship (Neneh, 2020). Vocational colleges also carry out various entrepreneurial education to teach students entrepreneurial knowledge and skills training to improve students’ entrepreneurial intention. However, students with high entrepreneurial intention still account for a very small number. According to the entrepreneurship report of Chinese college students, 7.7% of vocational college graduates choose flexible employment, of which only 3.1% choose entrepreneurship (Sciences, 2022). On the one hand, the reason for this phenomenon is that the entrepreneurial process is full of difficulties and uncertainties. A large number of data show that the failure rate of start-ups in 5 years is as high as 67% (Svotwa et al., 2022). On the other hand, it is due to the lack of experience and knowledge accumulation of entrepreneurs (Wach and Bilan, 2021). Therefore, a better understanding of the factors that affect individual entrepreneurial intentions can improve students’ entrepreneurial intentions so as to speed up the entrepreneurial process.

As a result of entrepreneurship as the primary impetus for the expansion of the economy, research in entrepreneurship is currently attracting a significant amount of attention from academics and researchers (Phraudomsitthinayok, 2019; Guerrero et al., 2020; Anjum et al., 2020c). In recent years, scholars have studied entrepreneurial intention from different perspectives (Wach and Bilan, 2021; Huang et al., 2022; Abdelfattah et al., 2022a). For example, Anjum et al. (2020b) explore the influence of perceived creativity disposition on entrepreneurial attitude and entrepreneurial intention based on the theory of planned behavior. In addition, it also analyzes the moderating mechanism of university support on perceived creativity disposition and entrepreneurial intention. Some scholars study entrepreneurial intention based on social cognitive theory. Neneh (2020) takes self-efficacy as a key predictive variable affecting entrepreneurial intention and discusses how entrepreneurial passion and social support affect entrepreneurial intention through entrepreneurial self-efficacy.

At the same time, numerous studies conducted recently concentrated on the ways in which individual characteristics, such as cognitive elements, can inspire entrepreneurial intention (Mahmood et al., 2018; Munir et al., 2019; Tariq et al., 2021). The cognitive elements enable people to identify and seize opportunities for entrepreneurship. In this context, the cognitive perspective of entrepreneurship sheds light on the importance of creativity. Researchers have acknowledged the significance of creativity as a factor in determining whether or not they would carry out entrepreneurial activities (Shi et al., 2020; Mickiewicz and Kaasa, 2022; Abdelfattah et al., 2022b). Literature indicates that a creative individual will most probably engage in entrepreneurial activities (Mahmood et al., 2018; Anjum et al., 2020b). However, further research is needed to determine whether creativity will affect the entrepreneurial intentions of students in vocational colleges.

The models of behavioral intention have been successfully utilized to predict future behavior for both managers and policymakers in social psychology research (Wach et al., 2016). In addition, the theory of planned behavior (TPB) constitutes the most frequently used model in the study of entrepreneurial intentions. The aim of this study is to explore the impacts of creativity on entrepreneurial intention by applying the TPB model. While other scholars have explored links between students’ creativity and entrepreneurial intentions, the innovations of this study are as follows: Firstly, creativity adopts a second-order formative construct and conceptualizes it into a multidimensional dimension (Zampetakis et al., 2011). This is because individual creativity is affected by the surrounding environment in the process of entrepreneurship, such as family and university (Rosique-Blasco et al., 2018). Secondly, this study has also emphasized how important it is to take into account the interactions between individual and environmental elements. For instance, the inclusion of social support in the entrepreneurial intention model adds a new dimension of exploration for the personal traits of entrepreneurs. Thirdly, since entrepreneurial activities are inherently risky, this study hypothesizes whether risk propensity (RP) moderates the relationship between creativity and entrepreneurial intention. Finally, relationships are examined between creativity and entrepreneurial intentions drawing on the TPB, both directly and as mediated by attitudes toward entrepreneurship and entrepreneurial self-efficacy.

This study focuses on answering the following research questions: (1) What are the entrepreneurial factors that affect vocational college students? And which factor has the greatest impact on entrepreneurial intention? (2) This study estimates the role of creativity and social support in the formation of entrepreneurial intentions. Are they direct antecedents of intentions, or are their effects mediated by attitudes and self-efficacy? (3) Sometimes, even people with high creativity still have no intention of participating in entrepreneurial activities. Can risk propensity help transform creativity into entrepreneurial intention?

The remaining portions of this article are described below. In the second section of this study, several hypotheses about the direct, indirect, and moderating effects of creativity and social support on entrepreneurial intention are justified and proposed. The third section lays down the methodology adopted to accomplish the stated research objectives. Next, we detail our findings based on information gathered from a representative sample of 500 students enrolled in vocational colleges. In the fifth part of this study, we summarize our findings as well as the implications for both theory and practice. The last section provides insights about the limitations and direction for future research.

## Literature review

### Theory of planned behavior

Ajzen (1991) put forward the theory of planned behavior on the basis of the theory of rational behavior (Ajzen and Fishbein, 1980). Multiple studies have applied theory of planned behavior (TPB) theory to analyze individual behavior in diverse relationships. For TPB, behavioral intention is the primary predictor that makes it easier to measure and assess behavior. According to TPB, three proximal dimensions that can influence behavior intention are the subjective norm, perceived behavioral control, and attitude. In order to more effectively explain behaviors, researchers frequently use the altered TPB model with the appropriate variable substitution or addition depending on each research scenario (Boudreaux et al., 2019). However, numerous scholars have found a non-significant direct relationship between subjective norms and entrepreneurial intentions (Esfandiar et al., 2019). This may be due to the fact that subjective norms have a marginal impact on the entrepreneurial intentions of individuals who possess great internal control (Icek Ajzen, 2002). Therefore, the variable is not considered in this study. Furthermore, perceived behavioral control is defined as the perceived ease or difficulty of performing a particular behavior (Ajzen, 1991). However, self-efficacy can take the place of perceived behavior control when explaining behavioral intentions (Armitage and Conner, 2001). Wach and Bilan (2021) argue that these two concepts are equivalent and reciprocal interchange. Therefore, we use self-efficacy to replace perceived behavior control in this research.

### Entrepreneurial intention

Entrepreneurial intention (EI), first proposed by Bird (1988), refers to the mental state of individuals when setting entrepreneurial goals. Scholars have had a long-standing interest in trying to understand the factors that contribute to entrepreneurial behavior. EI is the best predictor of entrepreneurial action as it precedes any attempt at entrepreneurial behavior (Anjum et al., 2020c). The study of EI is a rapidly developing area of research (Tariq et al., 2021; Wach and Bilan, 2021; Abdelfattah et al., 2022a). Research on EI can deepen people’s understanding of entrepreneurial cognition and behavior patterns. This study defines EI as individual’s psychological tendency to carry out entrepreneurial activities. It is the product of the interaction between individuals and the environment. Starting from personal personalities or the external environment, researchers have explored various factors that may lead to EI and studied its influence mechanisms, such as entrepreneurial personalities (Liu et al., 2021; Munir et al., 2021; Bui Ngoc Tuan and Pham, 2022), cognitive factors (Neneh, 2020; Mickiewicz and Kaasa, 2022; Abdelfattah et al., 2022b), environment elements (Wn et al., 2020; Hossain et al., 2021; Bui Ngoc Tuan and Pham, 2022). According to the findings of the researchers, these factors have an indirect impact on the intentions of individuals by influencing their attitudes or self-efficacy (Hossain et al., 2021; Liang et al., 2021; Westhuizen and Awotunde, 2021).

### Social support

Social support (SS) refers to obtaining substantial, emotional, and informational help from others when an individual is in need. It can be roughly categorized into three dimensions: emotional support; tangible support (e.g., financial assistance); and informational support (e.g., sharing valuable knowledge). Each of these three dimensions is described in detail. “Emotional support” (ES) is an intangible type of support received from one’s social network (Farooq, 2016). Taylor (2011) claimed that “emotional support” is a feeling of being cared for and a warm feeling of having positive recognition from one’s social network. The term “tangible support” (TS) is used by Farooq (2018) to characterize all of the concrete and direct ways in which people can help others, such as practical action and material support. According to Farooq (2016), more than half of all entrepreneurs give up on their business plans each year because they lack adequate resources and financial backing. Farooq (2018) described “information support” (IS) as an effective way to provide advice to entrepreneurs who are having trouble. If entrepreneurs do not have access to rich information or previous work experience, it is difficult for them to make accurate judgments (Farooq, 2016). As a result, obtaining informational help at the earliest stages of entrepreneurship is of the utmost significance for entrepreneurs (Markovic et al., 2017). These three factors are also taken into account by Huang et al. (2022) in a newly released study to evaluate the importance of SS and technology product imagination disposition in predicting individuals’ internet EI. Alzamel et al. (2020a) studied the impact of SS on females’ EI and the results showed that SS comprised of family and peers has a significant influence on entrepreneurial capability among females. Furthermore, Farooq (2018) considered that entrepreneurial behavior requires more support (such as financial support, human resources, information support, etc.) than any other behavior.

### Creativity (IC)

This study conceptualizes creativity as the driving force behind entrepreneurship as a way to explain how innovation promotes economic growth (Ng Kim-Soon and Mostafa, 2022). In the past, creativity was thought of as a characteristic of a person’s personality. However, recent research has highlighted the fact that creativity is the product of human interaction (Munir et al., 2021). Hence, the interplay between the environment and an entrepreneur can be a source of entrepreneurial creativity (Runco, 2014; Kusmintarti et al., 2017). For example, some studies highlight the role of the family in influencing individuals’ creativity and shaping EI (Zampetakis et al., 2011; Munir et al., 2021). Family creativity (FCRE) is thought as a personality feature, and it claims that an individual’s creativity is influenced by the environment in which they grow up (Zampetakis et al., 2011). Furthermore, the term “creative university environment” (UCRE) refers to the atmosphere at universities that fosters and encourages individual innovation (Amabile et al., 1996). Other scholars believe that educational environments have an impact on young people’s creativity. For instance, Muammar and Maker (2022) emphasize the influence of teachers’ characteristics and behaviors on students’ creativity. In addition, scholars agree that creative role models in school are conducive to influencing students’ creativity (Mickiewicz and Kaasa, 2022). The literature on creativity shows that creativity is an essential component of the entrepreneurial process (Anjum et al., 2018, 2020d; Rungsrisawata and Sutdueanb, 2019). For instance, Munir et al. (2021)‘s study is underpinned by the “personal abilities-intention based framework” by employing the integrated role of personal abilities (creativity and self-confidence) and the theory of planned behavior (TPB), finding a positive link between IC and EI. Mahmood et al. (2018) assert that entrepreneurs have a greater propensity for creativity than non-entrepreneurs do. Individuals with high creativity can maintain a positive attitude and high self-confidence toward entrepreneurial activities and think in novel and unconventional ways. Anjum et al. (2020b) introduce creativity into the EI model, estimating the positive relationship between perceived creativity disposition on entrepreneurial attitude and intentions.

### Risk propensity

It is inevitable that entrepreneurs will encounter unknown risks in the process of entrepreneurship. Risk propensity refers to the psychological characteristics of individuals who take the initiative to cope with or avoid risks (Sitkin and Weingart, 1995). The emotional and mental aspects of people who drive their entrepreneurial activities are highlighted in psychological theories of entrepreneurship. According to locus of control theory proposed by Julian Rotter in 1954, it manages to explain that entrepreneurs with internals locus believe that emergence of success is due to their capabilities and actions. Risk-taking is the most elementary action that entrepreneurs do to achieve high-level performance and success (Brockhaus, 1980). Bodill and Roberts (2013) points that individuals who have a high RP are more likely to choose high-risk entrepreneurial activities to achieve their goals because of the high profitability associated with such activities. Otherwise, they will give up entrepreneurship (Golik, 2018). In addition, the higher the individual’s risk tendency, the more likely it is to underestimate the risk in some cases, thereby reducing their perception of risk (Ward et al., 2019). Some literatures have introduced the variable of risk propensity to study the influencing factors of EI (Golik, 2018; Gu et al., 2018; Hussain et al., 2021). Gu et al. (2018) point out that individuals who make entrepreneurial choices have a higher risk preference than ordinary people. When facing the same entrepreneurial opportunities, they are more willing to pursue risks to obtain greater benefits and a sense of achievement and satisfaction. In addition, Hussain et al. (2021) propose that RP affects entrepreneurial self-efficacy and then affects the EI. At the same time, he considers that individuals with high-risk tendencies can obtain positive incentives even in an unknown environment, which has a positive impact on EI.

### Entrepreneurial self-efficacy

According to Chen et al. (1998), self-efficacy refers to a person’s belief in one’s own abilities to attain desired outcomes, which has a tremendous impact on an individual’s thoughts, feelings, and behavior. In the context of research on entrepreneurship, entrepreneurial self-efficacy (ESE) refers to the degree to which an individual believes in his or her skills and capabilities to successfully complete the duties necessary for entrepreneurship (H. Zhao et al., 2005; McGee et al., 2009). More and more attention is being paid to the importance of ESE in the prediction of EI. A variety of models have been extended and adjusted to incorporate self-efficacy as a major factor in determining whether or not an individual intends to engage in entrepreneurial activity (Ahmed et al., 2020; Urban, 2020; Liang et al., 2021; Westhuizen and Awotunde, 2021). For example, Sandi and Nurhayati (2020) took self-efficacy as a key antecedent to influencing EI and explored how factors such as entrepreneurship education and family environment affected the formation of EI by influencing ESE. The empirical results illustrated that self-efficacy has a significant impact on the interest of student entrepreneurship.

### Entrepreneurial attitude

“Entrepreneurial attitude (Ate)” refers to the general views of students on entrepreneurial behavior. It is a subjective judgment and an internal motivation for a person to choose to engage in entrepreneurship (Robinson et al., 1991). To a large extent, people’s attitudes determine whether or not they would engage in a particular behavior, as stated by Hgg and Gabrielsson (2019) (e.g., entrepreneurial activities in this study). When something is labeled “good for me,” it will produce a positive attitude, while when something is labeled “bad for me,” it will produce a negative attitude (Kusmintarti et al., 2017). Attitude, as an important determinant of intention, has appeared in many literatures (Wn et al., 2020; Anjum et al., 2020d, 2022). For example, Anjum et al. (2022) constructed the EI model by taking ATT as an important antecedent variable of EI based on the theory of planned behavior and pointed out that ATT has a significant impact on EI. The research results of Wn et al. (2020) also support this view; that is, students who have a positive attitude toward entrepreneurship are more likely to start businesses in the future.

## Hypotheses

### Social support and entrepreneurial intention

Previous studies identified SS as an essential determinant of EI (Kruse et al., 2020; Muhammed et al., 2020; Alzamel et al., 2020b; Hossain et al., 2021). For example, Hossain et al. (2021) consider that when entrepreneurs get good social support, they will be satisfied with their entrepreneurial environment and interpersonal relationships so as to stimulate their EI. Akter et al. (2020) discover that a socially supportive environment not only improves EI but also prepares the route toward for individuals who are just beginning their entrepreneurial journey. According to Wn et al. (2020), entrepreneurship is a social activity that calls for significantly more frequent interaction with one’s surrounding social environment than any other activity. As a result of these justifications, the following hypothesis can be posited:

*H1*: SS has a significant impact on EI.

### Social support and entrepreneurial self-efficacy

SS can enhance self-efficacy to a certain extent. Younis et al. (2020) hold that a lack of social support may contribute to increased anxiety and a lower sense of psychological well-being. This may be a vicious cycle. Neneh (2020) also proposes that SS can help entrepreneurs cope with the challenges in the process of entrepreneurship and enhance their sense of security and confidence, which in turn improves their self-efficacy. Hence, it is hypothesized in this study that:

*H2*: SS has a significant impact on ESE.

### Social support and entrepreneurial self-efficacy

Entrepreneurship is a complex process. Some scholars consider that the social support provided by the outside world to entrepreneurs can reduce the psychological anxiety of entrepreneurship. These supports can boost college students’ self-confidence and face the difficulties encountered in the future with an optimistic attitude. Therefore, social support can enable entrepreneurs to take a positive attitude toward entrepreneurial prospects (Anjum et al., 2022; Bakar et al., 2022; Bui Ngoc Tuan and Pham, 2022). On the basis of these findings, the following hypothesis is posited:

*H3*: SS has a significant impact on ATE.

### Creativity and entrepreneurial intention

There is a direct effect of creativity on EI. When people dedicate themselves to activities related to entrepreneurship rather than working for someone else, the returns from creativity are anticipated to be higher (Batchelor and Burch, 2012; Anjum et al., 2020a). A growing body of research underlines the relationship between entrepreneurship and creativity (Hu et al., 2018; Murad et al., 2021; Wang et al., 2021; Abdelfattah et al., 2022b). For example, the existing body of research backs up the assertion that creative individuals could have greater EI than uncreative individuals (Anjum et al., 2020d). Ndofirepi (2018) further demonstrates that different dimensions of creativity have diverse effects on EI. Murad et al. (2021) have indicated that the relationship between creativity and EI is indirect and will be influenced by a third factor. Based on these arguments, this study proposes the following hypotheses:

*H4*: IC has a significant impact on EI.

### Creativity and entrepreneurial attitude

The research conducted by Frank et al. (2007) demonstrates that personal characteristics (such as creativity) are the key to the origins of entrepreneurial behavior. People who possess a particular quality will likely feel more at ease engaging in activities that require that quality or imagining themselves conducting these activities (Muammar and Maker, 2022; Abdelfattah et al., 2022a). Thus, it seems that those individuals who are more creative have a more positive attitude toward entrepreneurship. For all these reasons, this study proposes the following hypothesis:

*H5*: IC has a significant impact on ATE.

### Creativity and entrepreneurial self-efficacy

As far as creativity is concerned, there is a growing literature examining relationships between creativity and ATE (Mahmood et al., 2018; Laguía et al., 2019; Anjum et al., 2020d). According to Mahmood et al. (2018), open-mindedness and the ability to break free of preconceptions are two of the characteristics of creativity that can help entrepreneurs gain creative confidence. For those with high creative confidence, self-efficacy will be higher and the possibility of entrepreneurial success will be greater. For this reason, the study chooses to test the following hypothesis:

*H6*: IC has a significant impact on ESE.

### Entrepreneurial self-efficacy and entrepreneurial intention

Perceived behavior control in TPB can be replaced by self-efficacy (SEF) when explaining behavioral intentions (Armitage and Conner, 2001). According to Akter et al. (2020), people with a high level of self-efficacy have great effectiveness and strategic flexibility in their entrepreneurial success, which will positively affect their EI and dare to bear the risk of entrepreneurial failure. In addition, an individual who has a high level of self-efficacy is better able to anticipate difficulties that can prevent them from achieving their specified goals (Westhuizen and Awotunde, 2021). In contrast to those who have low self-efficacy and believe they will constantly be wary of new environments, those who have a high level of self-efficacy are more likely to react positively to emerging situations (Li et al., 2020a,b). Indeed, several empirical studies have found self-efficacy to have a direct influence on entrepreneurial intention (Hsu et al., 2018; Ren et al., 2018; Neneh, 2020). So, it is hypothesized that:

*H7*: ESE has a significant impact on EI.

### Entrepreneurial attitude and entrepreneurial intention

According to Ajzen (1991), attitude refers to personal beliefs, which is the degree to which the individual likes or dislikes the outcomes that their behavior produces. From the perspective of students, ATE is the degree to which students desire or do not desire to engage in entrepreneurial behaviors. Many researchers have also explored the positive relationship between ATE and EI (Jena, 2020; Anjum et al., 2022; Bakar et al., 2022; Svotwa et al., 2022). The meta-analysis of entrepreneurship literature conducted by Hazarika et al. (2020) also indicates that ATE has the strongest predictive potential for EI, accounting for more than half of the total variance. Valencia-Arias et al. (2021) point out that although ATE is comprised of various motivations and varies depending on the individual, its impact on EI can be universal. Based on the literature, the following hypothesis is posited:

*H8*: ATE has a significant impact on EI.

### The role of the theory of planned behavior

According to the TPB, attitudes, subjective norms, and self-efficacy provide the most substantial information about a behavior’s determinants. Whereas other background factors like personality, demographics (such as age, gender, and education), and other information sources may indirectly affect EI through one or more of these elements (Ajzen, 2011). Accordingly, creativity and social support are expected to relate indirectly to EI through ATE and ESE.

#### Mediating effects of entrepreneurial self-efficacy

Students may have higher levels of ESE if they are more creative, which may lead them to believe that engaging in entrepreneurial behavior is simpler for them. If students are unable to see their own creative potential, it is possible that they may have less confidence in their ability to effectively complete such activities and view a career in entrepreneurship as being less attainable. Recent studies have shown that in addition to the direct effect of creativity on EI, this relationship is also mediated by self-efficacy (Bello et al., 2018; Mahmood et al., 2018; Kumar and Shukla, 2019). Kumar and Shukla (2019) identify creativity and active personality as the two leading factors of EI when studying the EI of management students. The results show that the impact of creativity on EI is partially mediated by ESE, which means that creativity does not completely affect EI. Similarly, Rosique-Blasco et al. (2018) and Laguía et al. (2019) also find self-efficacy as a significant determinant in mediating creativity and EI. Given the above discussion, the hypothesis is formulated as follows:

*H9a*: The relationship between IC and EI will be mediated by ESE.

Similarly, ESE has been selected as a potential mediator of SS and EI. Researchers argue that the support provided by universities or families enhances the self-efficacy of individuals, thereby improving their EI (Mei et al., 2017; Xu et al., 2020; Gm and Klk, 2021). In addition, Shi et al. (2019) recognize the role of ESE as a mediator of the positive relationship between SS and EI. Similarly, this result has been confirmed by more studies (Saeed et al., 2014; Schmitt et al., 2018; Neneh, 2020). Hence, the hypothesis is proposed:

*H9b*: The relationship between SS and EI will be mediated by ESE.

#### Mediating effect of entrepreneurial attitude

Krugger (2009) believes that the antecedents that influence EI are indirect. These antecedents first influence one’s attitude and then affect EI. In this study, antecedents include SS and CI. According to the results of several studies (Farrukh et al., 2018; Anjum et al., 2020b), creativity influences EI through attitude. Anjum et al. (2019) consider that students who are self-assured, capable of creative thought, and have a positive outlook on the possibility of starting their own business have a greater possibility of devoting themselves to the field. As a result, attitude can function as a proximal predictor of EI, whereas creativity can function as a more distant antecedent. The following hypothesis is offered:

*H9c*: The relationship between IC and EI will be mediated by ATE.

Farooq (2018) argues that SS has an indirect effect on EI of individuals. Namely, SS directly effects the ATE, which, later on, positively affects EI. Students can increase their positive attitude toward entrepreneurship and their recognition of their entrepreneurial identity with the help of the external environment. In addition, Younis et al. (2020) also show similar results: ATE plays a mediating role between SS and EI. Hence, this study hypothesis that:

*H9d*: The relationship between SS and EI will be mediated by ATE.

### Moderating effect of risk propensity

Some studies point out that RP moderates the relationship between creativity and EI (Gu et al., 2018; Samydevan et al., 2020; Hussain et al., 2021). In our model, this study views RP as the moderating mechanism relating creativity to EI. That is, people with higher risk propensity have higher creativity, and then have higher EI. Gu et al. (2018) propose that high-risk propensity individuals tend to adopt innovative ways to solve problems in the process of entrepreneurship, which is conducive to stimulating individual creativity and improving the success rate of entrepreneurship. Individuals with low-risk propensity tend to adopt traditional and error-prone methods to deal with difficulties, which inhibits the development of individual creativity and weakens individual EI. Based on the above analysis, the following hypothesis can be proposed:

*H10*: The relationship between IC and EI will be moderated by RP.

**Figure 1:**
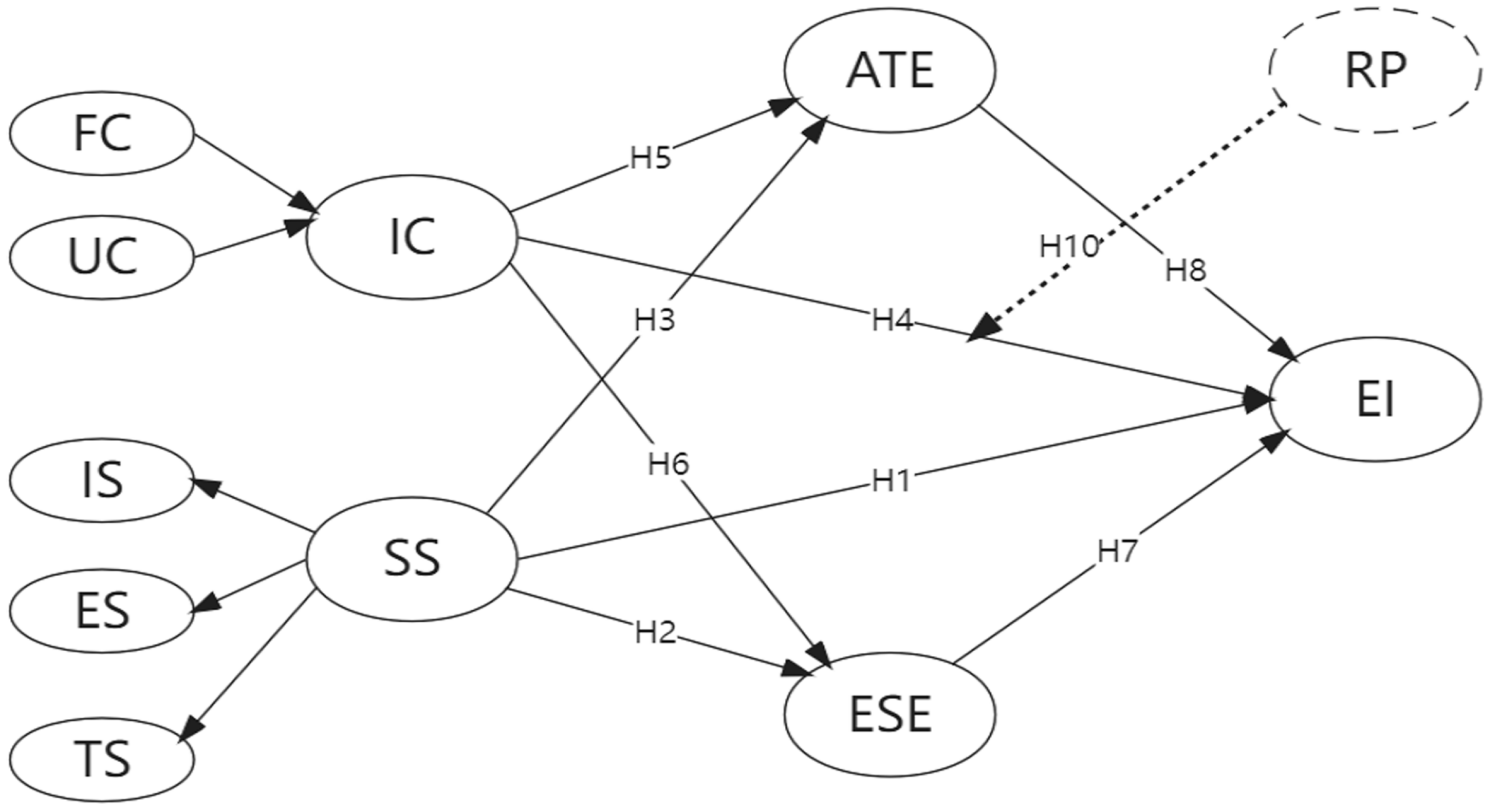
Conceptual framework.

A conceptual framework based on the theory of planned behavior is thus constructed on the basis of the above discussion and hypotheses. All hypotheses and latent variables are presented in the conceptual model in Figure 1.

## Materials and methods

### Participants

In this study, students from seven public and private vocational colleges in different provinces of China were selected as the samples. Online questionnaires were distributed through WeChat, QQ, email, and other platforms. The participants were asked to fill in a self-reported questionnaire designed to collect the data needed to test the theoretical framework proposed. The questionnaire is filled in anonymously to ensure its authenticity. Influenced by the policy, the government encourages vocational colleges to implement flexible educational systems, allowing students to adjust their academic process or suspend their studies for entrepreneurship. Therefore, this study is not only a survey of graduates but also a survey of students of all grades in vocational colleges and their intention to enter into entrepreneurship. The questionnaires were distributed and collected from June to July 2022. The random sampling method was used to proportionally distribute the questionnaires according to the actual number of students in each discipline. At the same time, the original samples were screened on the basis of accuracy and authenticity: on the basis of the number of questionnaires, the questionnaires were excluded if they were filled in less than 100 s. Ten consecutive questionnaires with the same answer were excluded. Finally, a list of 500 respondents was obtained, and 16 invalid questionnaires were eliminated, with a valid questionnaire rate of 96.90%. Therefore, the sample is representative and can effectively represent the characteristics of all students.

This study used SPSS26.0 to carry out a descriptive analysis of respondents demographic characteristics like gender, academic year, and major. From the data in Table 1, it can be concluded that there were slightly more males among the respondents, accounting for about 69.0% of the respondents; 31.0% of the respondents were women. The vast majority of them are students majoring in Social Sciences (47.2%) and freshmen (50.4%). Based on the recommendations of Hair (2009), the sample size should be 15–20 observations per variable for generalizability purposes. The total number of variables in this study was five. A sample size of more than 100 meets the requirements. The effective sample size collected in this study was 500. Therefore, the sample size was considered adequate (Hair et al., 2021).

**Table 1:** Demographic information of the sample.

Characteristics	Item	Frequency	Percentage
Gender	Male	345	69.0%
	Female	155	31.0%
Academic year	Freshman	252	50.4%
	Sophomore	165	33.0%
	Junior	83	16.6%
Major	Humanities major	187	37.4%
	Social science major	236	47.2%
	Natural science major	77	15.4%

### Measurement

The design of the questionnaire is a key element in the research process because it ultimately influences the collection of data (Sarstedt et al., 2019). This research questionnaire is divided into two sections. The first section consists of the respondents’ demographic profiles. The second section consists of independent variables and dependent variables. This study uses those as a basis for its measurements since earlier studies have developed accurate and reliable scales. To make it easier for respondents to convey their opinions, all responses are collected on a five-point Likert scale from 1 (strongly disagree), 2 (disagree), 3 (neutral), 4 (agree), and 5 (strongly agree).

The complete scale used in this study is shown in Table 2. Creativity is a second-order formative construct. In this study, it is classified into three dimensions: personal creativity (PCRE), creativity supported in the family (FCRE), and creativity supported in the university (UCRE). The three dimensions of creativity have different scales: The scales of FCRE are inherited from Miller and Gerard (1979) with three items for each scale; the UCRE is measured by three items that are adapted from Amabile et al. (1996); the survey items used to measure PCRE for this study have been adapted from Zhou and George (2001). Social support is a second-order reflective construct. It includes three first-order latent variables: informational support (IS), tangible support (TS), and emotional support (ES). To measure social support, this study used a scale modified from Krause and Markides (1990)’s study. The risk propensity construct, which has four items, is adapted from Anabela et al. (2013). Further, the ESE construct is measured with a four-item scale adapted from Zhao et al. (2005). There are five items for measuring EI that are adapted from Farrukh et al. (2019). Finally, a five-item scale of ATE adapted from Farrukh et al. (2019) is used.

**Table 2:** Survey instrument.

Construct		Item	Source
Creativity	Personal creativity	PCRE1: I think I am a very creative person.	Zhou and George (2001) and Sukendro et al. (2020)
		PCRE2: I like to try novel things despite the risk of failing.	
		PCRE3: I can easily think a lot of different and useful ideas.	
	Family creativity	FCRE1: My family members easily adapt to different circumstances.	Miller and Gerard (1979)
		FCRE2: My family members are always thinking about new ideas for making their life easier.	
		FCRE3: I can freely talk to my family members about new ideas.	
	University creativity	UCRE1: In my university you learn that there is more than one solution to a problem.	Amabile et al. (1996)
		UCRE2: In my university you learn to examine old problems in new ways.	
		UCRE3: In my university the faculty encourages students to produce new and useful ideas.	
Entrepreneurial attitude		ATE1: Being an entrepreneur would entail great satisfactions for me.	Farrukh et al. (2019)
		ATE2: I believe that if I were to start my business, I will certainly be successful.	
		ATE3: I’d rather be my own boss than have a secure job.	
		ATE4: A career as entrepreneur is attractive for me.	
		ATE5: Being an entrepreneur implies more advantages than disadvantages to me.	
Entrepreneurial intentions		EI1: I prefer to be an entrepreneur rather than to be an employee in a company or organization.	Farrukh et al. (2019)
		EI2: I will choose a career as an entrepreneur.	
		EI3: I intend to set up a company in the future.	
		EI4: I have a very serious thought in starting my own firm.	
		EI5: I will make every effort to manage my own firm.	
Entrepreneurial self-efficacy		ESE 1: I am convinced that I can successfully discover new business opportunities.	Zhao et al. (2005)
		ESE 2: I am convinced that I can successfully create new products.	
		ESE 3: I am convinced that I can think creatively.	
		ESE 4: I am convinced that I can successfully commercialize ideas.	
Social support	Tangible support	TS1: Supporters are willing to give me or lend me more than $1,000.	Krause and Markides (1990) and Moulang and Cahan (2015)
		TS2: Supporters give me or lend me what I need (materials and goods other than money).	
		TS3: Supporters are willing to help me do what I need to do, such as making a film.	
	Informational support	IS1: Supporters provide experience and tell me how to deal with problems in stressful situations.	
		IS2: Supporters suggest what I should do to solve the problem I am facing.	
		IS3: When I have a problem, a supporter provides me with information that helps to clarify the problem	
		IS4: Supporters help me understand why I’m not doing well.	
		IS5: When I have a problem, a supporter tells me whom to ask for help.	
		IS6: Supporters analyze the way I deal with the problem without commenting on its quality.	
	Emotional support	ES1: Supporters help me when I am under stress.	
		ES2: Supporters tell me that I can.	
		ES3: Supporters show empathy to comfort me.	
		ES4: Supporters listen to my inner feelings	
		ES5: Supporters show empathy when we have a heart-to-heart talk	
		ES6: Supporters try to cheer me up in a humorous and witty way.	
		ES7: Supporters express their attention and concern for my life	
		ES8: When I have a problem, a supporter will go with me to find someone who can help me.	
Risk propensity		RP1: I am willing to take high risks for high returns	Anabela et al. (2013)
		RP2: I do not mind working under conditions of uncertainty as long as there is a reasonable probability of gains from it for me.	
		RP3: I do not fear investing my money on a venture whose dividends I have calculated	
		RP4: I fear moving into a new undertaking I know nothing about.	

### Data analysis

In this study, the data is analyzed using variance-based partial least square-structural equation modeling (PLS-SEM) with the support of Smart-PLS version 3.0 software (Ringle et al., 2015). The technique is a powerful component-based method widely used in prior studies (Xu et al., 2020; Wang et al., 2021; Mickiewicz and Kaasa, 2022; Sciences, 2022). This method of estimation is suitable for this study for various reasons: (1) complex modeling including multiple mediators (Cepeda et al., 2018); (2) analyzing both reflective and formative measurement constructs in one model (David and Ketchen, 2013); (3) the estimation of formative measurement constructs without limitations (Henseler et al., 2009); (4) investigating complex relationships that combine mediating and moderating effects (Fornell et al., 1982); (5) second-order formative (i.e., creativity) and reflective (i.e., social support) multidimensional construct. A detailed description of the data analysis findings is given in the next section.

## Results

### The assessment of the measurement model

This study’s conceptual model includes both formative and reflective measurement constructs. Out of six total variables, one variable (i.e., creativity) is a formative measurement construct, and five variables (i.e., ATT, ESE, SS, RP, and EI) are reflective measurement constructs. Statistical evaluation criteria for reflective measurement constructs are different from formative measurement constructs (Hair et al., 2017). Accordingly, both reflective and formative measurement constructs are examined, respectively, in this study. Because a formative measurement scale’s items are likely to represent an independent cause and are not necessarily highly correlated with one another, the concept of internal consistency is inappropriate (Chin, 1998). Reflective measurement scale items must be correlated and should depict significant outer loadings (Hair et al., 2017).

#### Assessment of reflective measurement constructs

This study evaluates the internal consistency (reliability) and validity of constructs for the reflective measurement model. The internal consistency (reliability) is measured by the composite reliability (CR) and Cronbach’s alpha (CA). The value of CA shall be at least greater than 0.7. The CR values of 0.70 and 0.90 can be regarded as satisfactory. The CR values of more than 0.90 are deemed undesirable because this indicate “they are measuring the same phenomenon and are therefore unlikely to be a valid measure of the construct” (Hair, 2014). The results in Table 3 show all the values of CA and CR of the latent variables meet the requirements (Fornell and Larcker, 1981), indicating that the internal consistency of all the constructs is supported. The assessment of indicator reliability is done by testing the outer loadings of each indicator on its own latent variable, and the evaluated value is compared with the threshold value. The results in Tables 3, 4 demonstrate the reliability of the constructs involved in our proposed conceptual model.

**Table 3:** The reliability and validity of the first-order reflective constructs.

Construct	CA	CR	AVE
Family creativity	0.808	0.886	0.718
University creativity	0.878	0.898	0.764
Tangible support	0.958	0.874	0.843
Informational support	0.957	0.893	0.765
Emotional support	0.944	0.873	0.684
Entrepreneurial attitude	0.889	0.898	0.646
Entrepreneurial intentions	0.913	0.854	0.701
Entrepreneurial self-efficacy	0.945	0.887	0.713
Risk propensity	0.879	0.896	0.720

CA, Cronbach’s α; CR, composite reliability; AVE, average variance extracted.

**Table 4:** Reliability and validity of second-order reflective constructs.

Construct	CA	CR	AVE
Social support	0.976	0.881	0.752

A two-step approach is performed to validate the construct validity: the convergent validity and the discriminant validity.

It is required to test the convergent validity of the data to determine whether the items represent a particular construct (Urbach and Ahlemann, 2010). Convergent validity at the construct level is established using the average variance extracted (AVE; Fong and Law, 2013; Tehseen et al., 2017). As per the results, all values of AVE range from 0.646 to 0.843, which is acceptable and higher than the threshold critical level of 0.5 (Fornell and Larcker, 1981). Hence, its results demonstrate the convergent validity of reflective measurement models.

Discriminant validity is the extent to which a construct is truly distinct from other constructs (Hair, 2014). The test for discriminant validity of reflective constructs is performed by evaluating the cross-loadings of constructs’ indicators (Hair Hult et al., 2016). For the cross-loadings, indicators should have the highest loading on their own latent construct as compared to other variables involved in the model. Since the model in this study contains second-order constructs, the cross-loading of first-order reflective constructs (IS, ES, TS) is tested first. Then, the cross-loading of structural model constructs is calculated. The complete list of cross-loadings of all indicators of each construct is presented in Tables 5, 6. Hence, these findings meet the cross-loadings evaluation criteria and provide satisfactory evidence for the discriminant validity of the reflective measurement models.

**Table 5:** The cross-loadings of first-order construct (social support).

	ES	IS	TS
ES1	**0.894**	0.853	0.737
ES2	**0.899**	0.855	0.746
ES3	**0.903**	0.787	0.687
ES4	**0.917**	0.780	0.713
ES5	**0.906**	0.781	0.715
ES6	**0.898**	0.745	0.672
ES7	**0.913**	0.803	0.717
ES8	**0.919**	0.801	0.704
IS1	0.796	**0.898**	0.831
IS2	0.790	**0.899**	0.771
IS3	0.781	**0.901**	0.786
IS4	0.784	**0.905**	0.782
IS5	0.810	**0.897**	0.759
IS6	0.825	**0.925**	0.747
TS1	0.713	0.745	**0.932**
TS2	0.753	0.819	**0.941**
**TS3**	0.711	0.839	**0.933**

Bold values are outer loadings for each construct.

**Table 6:** The cross-loadings of construct.

	ATE	EI	ESE	IC	SS
ATE1	**0.741**	0.572	0.466	0.598	0.464
ATE2	**0.789**	0.567	0.571	0.641	0.516
ATE3	**0.827**	0.703	0.580	0.443	0.520
ATE4	**0.875**	0.756	0.707	0.543	0.646
ATE5	**0.869**	0.779	0.685	0.465	0.648
EI1	0.717	**0.819**	0.615	0.456	0.561
EI2	0.743	**0.901**	0.751	0.517	0.665
EI3	0.743	**0.923**	0.750	0.556	0.698
EI4	0.712	**0.904**	0.786	0.559	0.703
EI5	0.638	**0.802**	0.761	0.590	0.748
ESE1	0.708	0.814	**0.926**	0.571	0.751
ESE2	0.700	0.805	**0.940**	0.549	0.747
ESE3	0.680	0.784	**0.939**	0.574	0.780
ESE4	0.717	0.813	**0.935**	0.594	0.766
FCRE	0.635	0.570	0.767	**0.932**	0.566
UCRE	0.578	0.514	0.551	**0.879**	0.604
ES1	0.602	0.673	0.700	0.565	**0.883**
ES2	0.608	0.672	0.733	0.565	**0.894**
ES3	0.572	0.640	0.663	0.533	**0.859**
ES4	0.604	0.634	0.658	0.562	**0.870**
ES5	0.601	0.657	0.649	0.549	**0.864**
ES6	0.621	0.642	0.638	0.529	**0.843**
ES7	0.599	0.642	0.683	0.525	**0.880**
ES8	0.603	0.664	0.636	0.556	**0.877**
IS1	0.621	0.702	0.760	0.557	**0.880**
IS2	0.608	0.683	0.726	0.553	**0.866**
IS3	0.606	0.691	0.731	0.553	**0.865**
IS4	0.547	0.624	0.667	0.525	**0.867**
IS5	0.535	0.654	0.662	0.569	**0.873**
IS6	0.613	0.691	0.731	0.549	**0.914**
TS1	0.622	0.703	0.734	0.562	**0.821**
TS2	0.623	0.722	0.736	0.554	**0.853**
TS3	0.645	0.723	0.742	0.612	**0.847**

Bold values are outer loadings for each construct.

As can be observed, all of the requirements for model evaluation have been satisfied, which lends support to the model’s reliability and validity. Then, the discussion continues with the assessment of formative constructs involved in this study.

#### Assessment of formative measurement constructs

Reflective-formative higher order construct modeling is widely used in the PLS-SEM approach. According to Sarstedt et al. (2019), reflecting-formative type models are frequently specified using a two-stage approach. First, it gets the latent variable score (LVS) by estimating the first-order dimensions without the second-order construct in the model. Then, in the second stage of analysis, these LVS are used to measure the second-order formative construct (Wilson, 2010; Jan-Michael et al., 2012). This study modeled “creativity” as a reflective-formative construct; it has three lower-order constructs (LOC): PCRE, FCRE, and UCRE.

Formative constructs are evaluated differently than reflective constructs (Chin, 1998; Leguina, 2015; Henseler et al., 2016). All formative measurement constructs are likely to indicate an independent cause for the latent variable and have a low correlation among measurement scale items. Consequently, for the formative measurement constructs, this study analyzes the convergent validity, collinearity issues, and the weights of the formative indicators.

**Figure 2:**
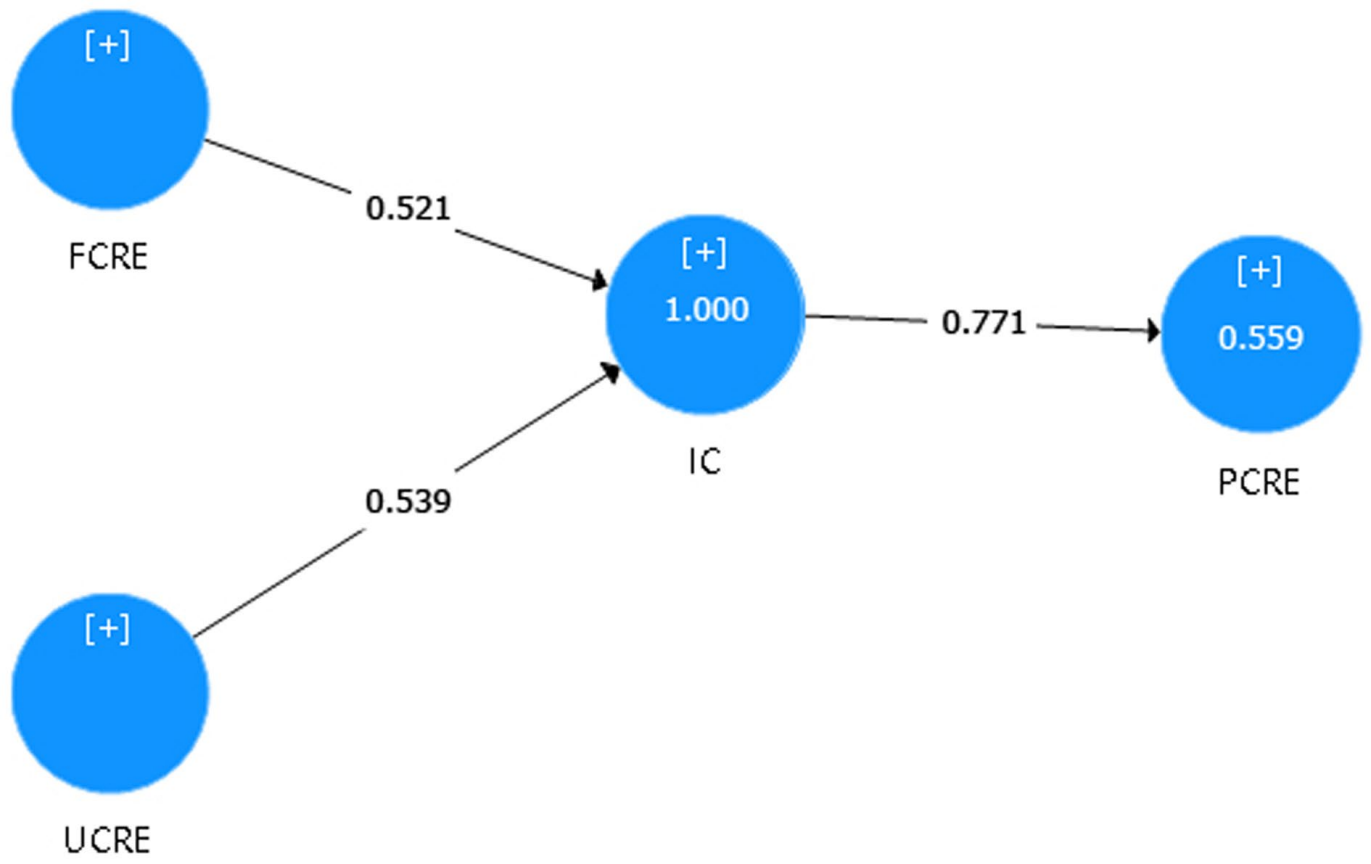
Convergent validity of second-order formative construct.

In formative measurement constructs, the computation for convergent validity is also performed differently (Chin, 1998; Leguina, 2015). As mentioned in the previous section, this study involves a formative construct (i.e., creativity). In order to establish convergent validity, this study has to test whether the formative construct is highly correlated with a reflective measure of the same construct (i.e., IC^formative^ → IC^reflective^). According to the rule of thumb, ideally, the correlation value between Y ^formative^ → Y ^reflective^ should be 0.80 or higher for determining the convergent validity of the formative construct (Chin, 1998; Leguina, 2015). Results in Figure 2 demonstrate that IC^formative^ → IC^reflective^’s path coefficient value is 0.771, close to the threshold of 0.80, which is acceptable for this study. Thus, the formative measurement construct (i.e., creativity) has an acceptable degree of convergent validity.

An important concern with formative construct is the level of multi-collinearity across formative sub-dimensions. Collinearity refers to the high correlation between two formative indicators. High levels of collinearity between formative indicators are a crucial issue because they have an impact on the estimation of weights and their statistical significance. Validation of variance inflation factor (VIF) values, which should be lower than 5, is used to check the collinearity issue of constructs (Hair and Sarstedt., 2011). The results in Table 7 indicate that all VIF values are less than 5. Therefore, the collinearity issue is not present between constructs.

**Table 7:** Collinearity test.

	ATE	EI	ESE	IC	SS
ATE		2.547			
EI					
ESE		3.554			
IC	1.516	2.044	1.516		
SS	1.516	3.390	1.516		

Outer weights in formative measurement models should be analyzed for their significance and relevance only if collinearity is not at a critical level (Diamantopoulos et al., 2008). The weight is similar to the path coefficient and explains the effect of each dimension on the formative construct. The significance of the weights confirms the significance and relevance of the multidimensional construct of creativity. Findings in Table 8 demonstrate that the weights of all the two indicators of creativity are significant. Hence, the significant weights made it possible for creativity to have more than one dimension and be studied further.

**Table 8:** Formative indicators weights, significance and test of multi-collinearity.

	First-order constructs	Weights	t-statistics (|O/STDEV|)	*p*-values	VIF
IC	FCRE	0.632	63.812	0.000^***^	2.590
	UCRE	0.440	54.273	0.000^***^	2.590

FCRE, Family creativity; UCRE, university creativity.

*** *P* < 0.001.

Based on what we have talked about so far, it is also clear that the applicability of formative constructs has also been established, and the overall assessment of formative measurement models has shown acceptable results to proceed with the evaluation of the structural model.

### Assessment of the structural model

The results of the evaluation of the measurement models show that all the reflective and formative measurement models are reliable and valid. For the structural model, this study analyzes the significance of the path coefficients, collinearity assessment, coefficient of determination(R^2^), the predictive relevance (Q^2^), and absolute model fit indices.

#### Collinearity assessment

The same measure used in the evaluation of formative measurement models is used in this study to evaluate collinearity. As per the rule of thumb, if the variance inflation factor (VIF) values in the study model are less than 5, it indicates that the study model does not have a covariance problem (Hair et al., 2017). Table 7 shows that the variance inflation factor (VIF) values for all the constructs range from 1.516 to 3.554, which is below the suggested threshold value of 5. This means that the model estimates do not have a multicollinearity bias and the results of the study model are relatively stable.

#### Coefficient of determination (R^2^)

The coefficient of determination (R^2^) is a measure of the model’s predictive accuracy and represents the exogenous latent variables’ combined effects on the endogenous latent variable. The R^2^ is classified as 0.25 (weak), 0.50 (moderate) and 0.75 (substantial; Hair Black et al., 2010). The R^2^ values shown in Table 9 range from 0.578 to 0.739, indicating that the model’s fit is satisfactory. The present study has an R^2^ value of 0.739 for EI, which demonstrates that the dependent variable is influenced by the independent variables by 73.90%. The R^2^ values indicate that the proposed conceptual model has adequate explanatory power.

**Table 9:** Coefficients of determination (R^2^).

Construct	R^2^ value
ATE	0.578
EI	0.739
ESE	0.660

#### Predictive relevance (Q^2^)

According to Hair et al. (2017), it is not sufficient to evaluate the predictive accuracy of the model only based on the R^2^ value. Hence, this research uses the Stone-Geisser’s (Geisser, 1974; Stone, 1974) Q^2^ test for evaluating the predictive relevance of a structural model by using the blindfolding procedure. If the Q^2^ value is larger than zero, it indicates that the structural model’s latent exogenous constructs have predictive relevance for latent endogenous constructs (Chin, 2010). As shown in Table 10, Q^2^ values range from 0.355 to 0.620, indicating all latent underlying endogenous constructs involved in this study have strong predictive relevance.

**Table 10:** Predictive relevance (Q^2^).

Construct	Q^2^ value
ATE	0.355
ESE	0.580
EI	0.620

#### Absolute model fit indices

A good-fitting measurement model is necessary before interpreting the structural model’s causal paths. The fit of the model is evaluated using the standardized root mean square error (SRMR) in this study. The SRMR is an absolute measure of fit, which is defined as the standardized difference between the observed correlation and the predicted correlations (Henseler et al., 2016). A good fit is generally considered to be less than 0.08 (Hu and Bentler, 1999). The SRMR value is 0.065 in this research, which is lower than 0.08. Consequently, the fitting degree of the model is acceptable (Table 11).

**Table 11:** Model fit.

Criteria	Acceptable value/condition	Actual value
SRMR	<0.08	0.065

#### Hypotheses test

The analysis of the measurement model and the structural model indicates that the proposed theoretical model is fit to proceed with hypothesis testing. Path coefficient values and t-values suggest different levels of support for hypotheses proposed in the structural model of this study (David and Ketchen, 2013).

Findings of the structural model demonstrate that, except a comparatively weak negative of creativity and EI, all other hypothesized path relations are positive and significant. H1 predict a positive relationship between SS and EI, hence supported at *p* < 0.001. The relationship between SS and ESE (H2) is also accepted (*p* < 0.001). Hypothesis relation between SS and ATE (H3) is found to be fairly significant and positive (β = 0.444; *t*-value = 6.938). Hypothesis 4 predicts a significant relationship between IC and EI and accepted at *p* < 0.05. The result prove that H5 propose a significant association between IC and ATT (β =0.371; *p* < 0.001) and it is supported by the dataset. A similar level of support is found for H6 (IC → ESE), describing a significant and positive relationship (β = 0.142; *t*-value = 2.840) between IC and ESE. ESE is found to have strong effect on EI (β = 0.434; *p* < 0.001). Thus, H7 is supported. Finally, ATE has a more important role than ESE in enhancing the EI (β = 0.471 > 0.444).

### Mediation analysis

Mediation is the process in which a variable or variables influence other variables through intervening or moderating variables (Hayes, 2013). Albers (2010) considers that the evaluation of the mediating influence should include both direct and indirect effects. To better illustrate the mediation effect, it is calculated using the bootstrapping method. It is possible to apply bootstrapping to small sample sizes because it does not infer the sampling distribution of statistical data or the distribution of variables (Hair et al., 2017). Therefore, for the PLS-SEM method, a bootstrapping strategy to test the indirect effects is very suitable.

**Figure 3:**
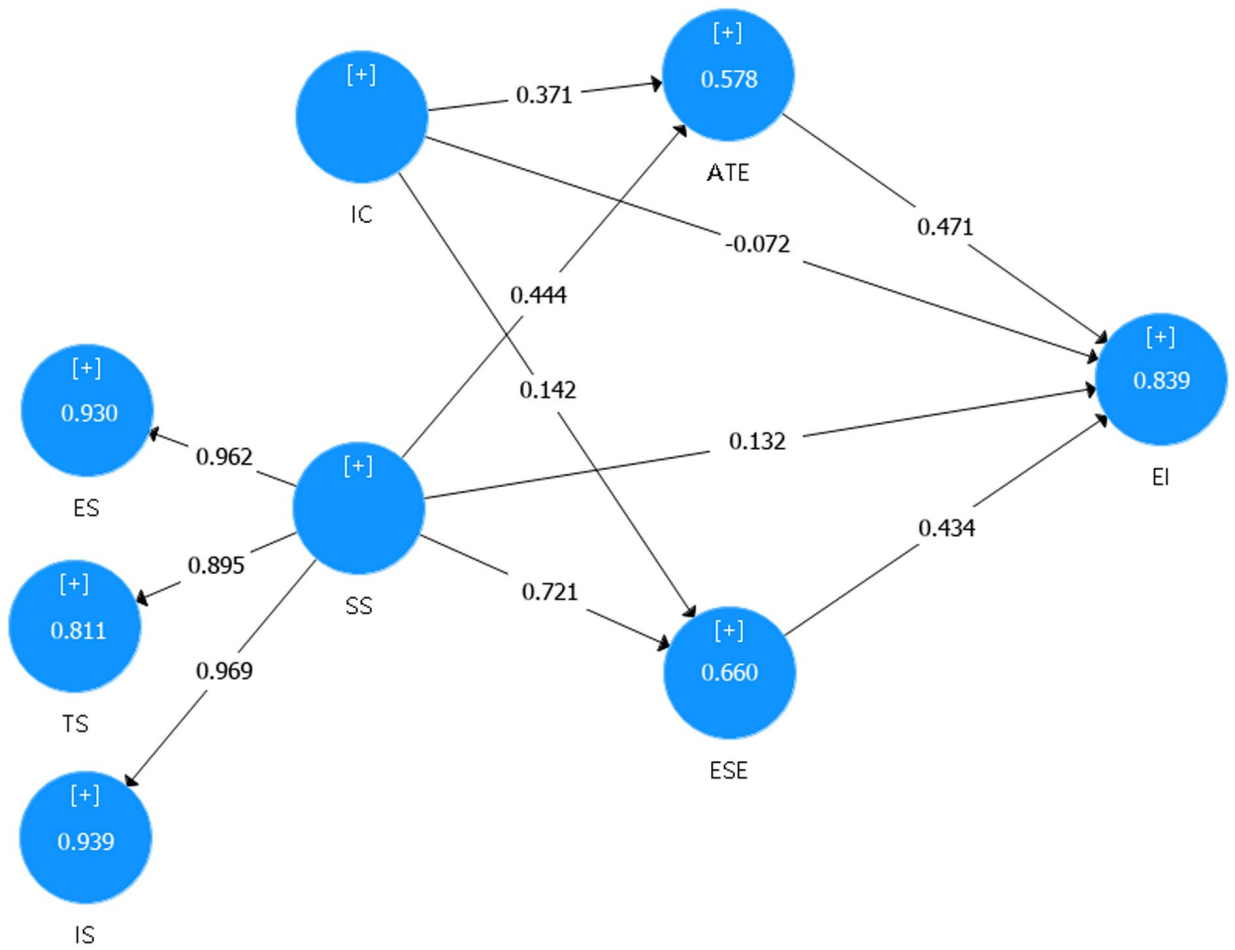
PLS results of the research model.

Mediation expects to meet the following conditions: (a) Analyze the significance of the direct impact without considering the mediator variable. It can be seen from Figure 3 that creativity has a significant effect on EI (β = −0.072, *p* < 0.05); There is a significant positive correlation between SS and EI (β =0.132, *p* < 0.01). (b) If the direct effect is significant, introduce the mediator variable in the PLS path model and assess indirect effect. (H9a, H9b, H9c, H9d). The results show in Table 12 that creativity and ESE (H6), creativity and ATE (H5); ESE and EI (H7), ATE and EI (H8); SS and ESE (H2), SS and ATE (H3); The relationship between them is significantly positive correlation. (c) If the indirect effect is significant, the mediator absorbs some of the direct effect (Hair, 2014). (d) The degree of absorption of mediator variables is evaluated by variance accounted for (VAF), which is calculated through the following formula: VAF = Indirect effect/total effect * 100, Total effect = indirect effect + direct effect. If VAF is less than 20%, there is no mediation effect; If the VAF is larger than 20% and less than 80%, that can be characterized as partial mediation; full mediation exist if the VAF is above 80%.

**Table 12:** Path analysis and hypothesis testing (first model).

Hypotheses	Path coefficient	Standard error	*T*-value	*P*-value	Decision
H1	0.132	0.043	3.070	0.002^**^	Supported
H2	0.721	0.052	13.865	0.000^***^	Supported
H3	0.444	0.064	6.938	0.000^***^	Supported
H4	−0.072	0.036	2.000	0.041^*^	Supported
H5	0.371	0.058	6.397	0.000^***^	Supported
H6	0.142	0.050	2.840	0.005^**^	Supported
H7	0.434	0.053	8.189	0.000^***^	Supported
H8	0.471	0.051	9.235	0.000^***^	Supported

* p < 0.05;

** *p* < 0.01;

*** *p* < 0.001.

There will be an exception to the VAF-based mediation effect assessment, namely the suppressor effect (Hair, 2014). For example, the VAF becomes larger than one or, in some instances, even negative with suppressor effects and can no longer be interpreted. This kind of situation always represents full mediation. Therefore, the results in Table 13 show that ATE plays a complete mediating role in IC and EI because the value of VAF is negative; the ESE also plays a complete mediating role in IC and EI because the value of VAF is greater than 1 (Hair, 2014). In addition, the ESE accounts for 70.7% of the effect of SS on EI, indicating partial mediation; the ATT can account for 62.3% of the effect of SS on EI, which is called partial mediation.

**Table 13:** The result of mediation analysis.

Independent variable	Mediator variable	Dependent variable	Direct effect	Indirect effect	Total effect	VAF	Hypothesis
Individual creativity	Entrepreneurial self-efficacy	Entrepreneurial intention	−0.072 (2.021)	0.062 (2.910)	−0.01	−6.20	Supported (H9a)
Individual creativity	Entrepreneurial attitude		−0.071 (2.021)	0.173 (4.729)	0.102	1.696	Supported (H9c)
Social support	Entrepreneurial self-efficacy		0.132 (2.972)	0.320 (6.545)	0.452	70.7%	Supported (H9b)
Social support	Entrepreneurial attitude		0.132 (2.972)	0.218 (6.270)	0.350	62.3%	Supported (H9d)

### Moderation analysis

Moderation is defined as a condition in which the relationship between two constructs is not constant but depends on the value of a third variable known as the moderator variable. The moderator variable affects the intensity or direction of the interaction between the two structures. Hair (2014) points out that the product indicator method cannot be used when either the exogenous latent variable or the moderator variable has a formative measurement model. Hence, this study use the two-stage approach by making explicit use of PLS-SEM’s advantage to estimate latent variable scores (Henseler and Chin, 2010; Rigdon et al., 2010).

Table 14 illustrates the results of the moderation analysis that indicate that RP moderates the IC-EI relationship. Furthermore, the moderation results for path coefficient, t-value, and value of p showed a significant relationship. In other words, when the moderator variable increases by one standard deviation, the slope of creativity (the independent variable) to entrepreneurial intention (the dependent variable) will increase by 2.239 standard deviation.

**Table 14:** The result of moderation analysis.

Hypothesis	Path coefficient	*T*-statistics	*P*-value
H10	0.097	2.239	0.031

## Discussion

**Figure 4:**
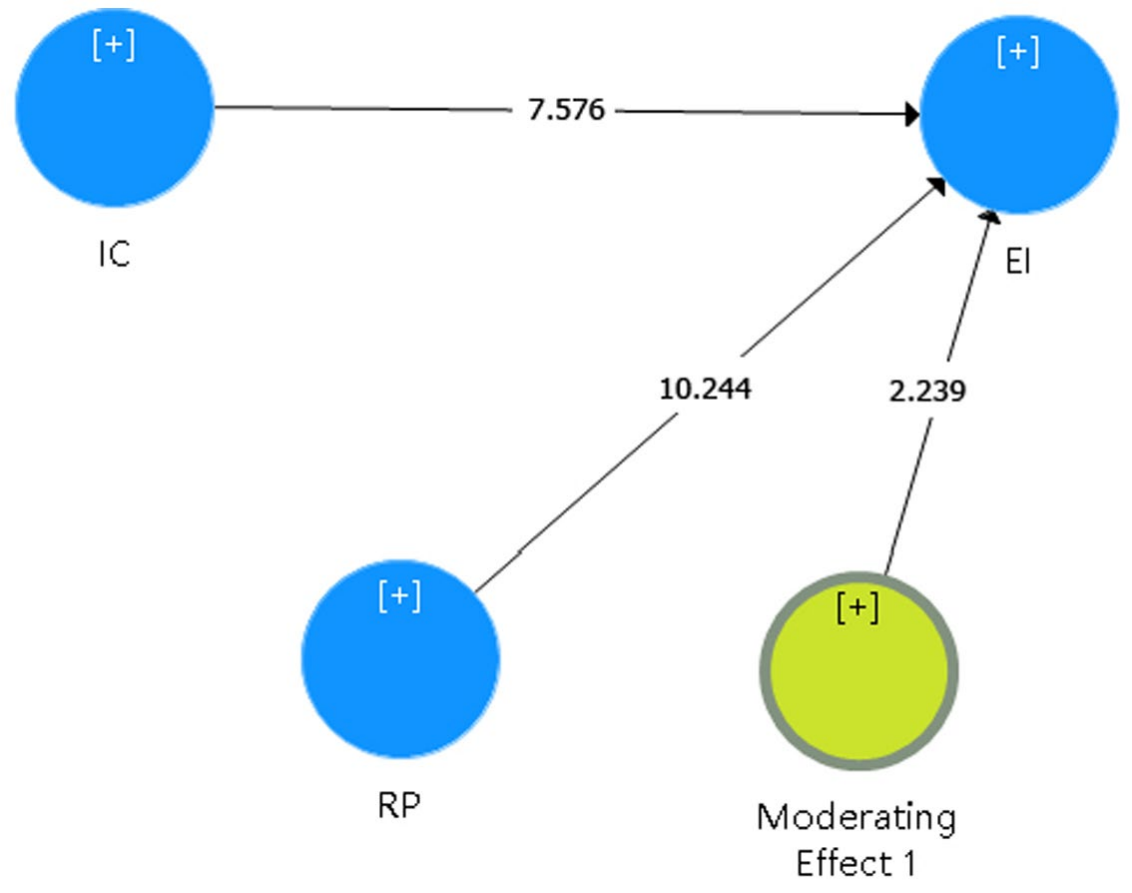
The moderating role of risk propensity.

The unprecedented high unemployment rate makes entrepreneurship an option for fresh graduates with the impact of the COVID-19 and a sharp increase in employment pressure. Therefore, the purpose of this study is to understand the underlying mechanism behind the formation of entrepreneurial intentions based on the theory of planned behavior. Furthermore, this study hypothesizes that entrepreneurial intention is predicted by four variables: creativity (IC), social support (SS), entrepreneurial attitude (ATE), and entrepreneurial self-efficacy (ESE). Meanwhile, the moderating variable between creativity and entrepreneurial intention is risk propensity (RP). The research model presented in Figure 4 is validated using PLS, and the results showed a good fit to the data. The hypothesized relationships among the constructs from the research model are supported.

The findings, which are based on the TPB framework, are validated among students attending vocational colleges in China. The two proximal attitudinal dimensions of TPB significantly predict EIs among students and confirm the predictive power of the model, accounting for 83.9% of the total variance in the student’s EI. The most important factor for predicting EIs for the current study is ESE, which is consistent with the previous studies by Laguía et al. (2019), Li et al. (2020a), and Li et al. (2020b) The research questions raised in the first paragraph of the introduction are discussed in the next few paragraphs. All predictors (SS, ATE, ESE) have a significant and direct relationship with entrepreneurial intention (EI), while creativity has an indirect effect on EI through entrepreneurial self-efficacy and attitude. From Figure 4, these indirect relationships can be shown: SS → ATE→EI; SS → ESE → EI; IC → ATE→EI; and IC → ESE → EI.

The SS significantly predicts EI (H1) among students and confirms the predictive power of the model. The empirical findings of this study are in line with Farooq (2018). Farooq (2018) explored the role that SS and entrepreneurial skills play in individual EIs using the theory of planned behavior. The findings indicate that SS and entrepreneurial skills have a positive and significant impact on EI. From the perspective of social support, family and school are the main places for students to socialize. The support of “important groups” such as teachers, relatives, friends, and classmates will affect college students’ behavioral intentions. Entrepreneurship is a social activity. Choosing entrepreneurship is a major decision in college students’ career planning. Therefore, college students will seek advice and support from surrounding people, and the degree of support from important groups will affect individual EI. Therefore, it is very important to formulate policies to encourage students to start businesses so that they can get support and build confidence in the process of entrepreneurship.

SS influences ESE directly and significantly (H2). The findings support the hypothesis proposed in earlier studies (Khayru et al., 2021; Bui Ngoc Tuan and Pham, 2022). Bui Ngoc Tuan and Pham (2022) found that ESE is proportional to the amount of SS an individual receives from the surrounding environment. The more support an individual receives, the higher their level of self-efficacy will be. Governments at all levels have provided policy and material support for students’ entrepreneurship. At the same time, the innovation and entrepreneurship courses offered by vocational colleges have expanded students’ knowledge of entrepreneurship. This reduces the difficulty of students’ entrepreneurship to a certain extent, thereby improving the self-efficacy of students’ entrepreneurship.

There is a direct relationship between the SS and ATE (H3). Consistent with the findings obtained in previous studies (Anjum et al., 2020a; Valencia-Arias et al., 2021). Bui Ngoc Tuan and Pham (2022) reported that the assistance provided by the outside world to the entrepreneurs themselves can reduce the difficulties in starting a business and thus have a positive attitude toward entrepreneurship. All attitudes, including entrepreneurial attitudes, can change. It is possible to drive the positive emotions of students to participate in entrepreneurial activities and form a positive attitude through the support provided by the government and schools to college students’ entrepreneurial activities.

Creativity has a statistically significant direct effect on ATE (H5). The result is in line with the outcomes of the study conducted by Anjum et al. (2020d). He empirically concluded that the association between creativity and attitude was significant using a sample of university students. This suggests that individuals with high creativity can produce more innovative ideas to solve problems in the process of entrepreneurship. They dare to meet unknown challenges and have a positive attitude toward entrepreneurship.

As expected, the results of the present study point out that there is a direct and positive relationship between creativity and ESE (H6). Other studies have also empirically confirmed this relationship (Shi et al., 2019; Ahmed et al., 2020). A study conducted by Kumar and Shukla (2019) among management students finds that creativity is also considered to influence ESE significantly. Individuals with high creativity can break through the original thinking framework to think about problems, produce more innovative ideas, and have a higher level of confidence in their own ability to succeed as entrepreneurs.

The present empirical study finds through data analysis that ESE is the most influential predictor of EI (H7). Some previous researchers also found the same findings (Malebana and Swanepoel, 2014; Akter et al., 2020). Among other factors, Akter et al. (2020) identified that ESE actually plays the most important role in explaining EI. This could be due to the fact that self-efficacy is one of the most important personality traits, and it helps individuals become more capable of overcoming challenges and work in adverse situations. In addition, ESE itself represents their confidence in entrepreneurial success, so promoting self-efficacy is a powerful force to promote individual entrepreneurship.

This study demonstrates the significant role of attitude in EI (H8); that is, attitude is an important factor in determining whether an individual has intention. This result is consistent with Temoor Anjum et al. (2022) who found that ATE directly impacts EI as well as significantly mediates the relationship between entrepreneurial education and EI among business students. It demonstrates that the individual’s evaluation of the results of entrepreneurial activity has a direct correlation to the individual’s ATE, which in turn has a direct correlation to the individual’s EI.

In line with the prior literature (Biraglia and Kadile, 2017; Mahmood et al., 2018; Ndofirepi, 2018), the relationship between creativity and EI is completely mediated through ESE (H9a) and the variance explanation of creativity on EI is less than 20%. Hence, creativity, as a factor in influencing entrepreneurship, is a low predictor. Also, the results from the study of Rodrigues et al. (2019) indicate that there is a weak relationship between creativity and EI in university students. This shows that creativity is not under an individual’s complete volitional control in many situations. Individuals with high creativity can guide entrepreneurs to set challenging goals, improve their ability to solve problems, and then enhance ESE. Vocational college students with creative characteristics not only show a higher sense of ESE but also are more sensitive and concerned about entrepreneurial information and entrepreneurial opportunities. Therefore, they may be more likely to have EI.

The empirical results show that self-efficacy mediates the links with social support and intentions (H9b), which consist of several studies (Neneh, 2020; Westhuizen and Awotunde, 2021). Neneh (2020) confirmed that the relationship between SS and entrepreneurial passion and EI is partially mediated by ESE using a sample of high school students. When individuals are provided with a greater quantity of positive feedback from their surroundings, it can assist them in reducing the psychological anxiety associated with being an entrepreneur and increasing their confidence in their ability to entrepreneurship. As a result, it will contribute to an improvement in one’s sense of self-efficacy.

ATE plays a completely mediating role between creativity and EI (H9c), which is in line with the previous studies (Hu et al., 2018; Rodrigues et al., 2019). Anjum et al. (2020d) examined the mediating role of attitudes to enhance the creativity disposition toward EI and found that attitudes have a significant mediating role. The variance explanation of creativity on EI is less than 20% in this study, so the direct effect of creativity on EI is weak. This means that when students’ creativity can increase their positive attitude toward entrepreneurship, their EI will increase. Students who are able to propose novel ideas and help solve problems are more inclined to start a business because they like flexible working hours and are willing to work hard for entrepreneurship.

According to the results, SS is a key factor in enhancing EI. Nonetheless, this link is indirect, mediated by attitude (H9d). These results corroborate and are in line with the previous research studies (Murad et al., 2021; Bui Ngoc Tuan and Pham, 2022). Hence, not only should the government and schools offer extraneous support in the form of funds, policies, but they should also pay attention to strengthening positive incentives for students, guiding students to make positive attributions to failure, cultivating students’ optimism, and finally promoting the improvement of students’ EIs.

RP positively moderates the relationship between IC and EI (H10).This result is consistent with Hussain et al. (2021) who believe that the relationship between creativity and EI is regulated by RP because risk is inevitable in the process of entrepreneurship. In the entrepreneurial literature, it is not uncommon to regard personal characteristics as factors that may affect the entrepreneurial process (Phraudomsitthinayok, 2019; Liu et al., 2021; Tariq et al., 2021). In most cases, however, only their primary effects as exogenous variables are taken into consideration when developing a causal model, and this study takes risk propensity as the moderating variable of creativity and EI. The moderating effect is mainly reflected in the following two aspects: On the one hand, individuals who have a high curiosity are willing to take risks and try new things, which contributes to the significance of the impact that creativity has on EI. On the other hand, people who have a high-risk propensity typically believe that “high risk, high return.” have the courage to take on unknown challenges, and their creativity is improved in this process.

## Conclusion

It is of great value to improve students’ EI to ease the employment pressure caused by the spread of the global epidemic. Thus, the current research is to examine the elements that influence vocational college students’ EI based on the theory of planned behavior. This study empirically tested the intention framework on a sample of 500 vocational college students. According to the findings of the data analysis, the 13 hypotheses of this study have been supported and verified to varying degrees. The results show that SS directly affects EI; students’ CI and SS are positively related to ATE and ESE, which are subsequently related to students’ EI. However, CI is not directly related to EI, which needs to be completely mediated by ATE and ESE. This is an important finding which helps us to understand why creativity has a less direct effect on students’ EI. In addition, this research examines risk propensity as a psychological mechanism that plays a moderating role in the association between creativity and EI. Overall, the proposed model fit well and explained 83.9% of the variance, which demonstrates that the proposed model can predict and explain the factors that affect students’ EI to a certain extent.

## Implications

### Practical implications

The results of this study show that creativity is crucial to improving the EI of vocational college students. At the same time, it also has some enlightenment for educational institutions and government departments.

Firstly, it is generally agreed that universities are the best places to learn new knowledge, ideas, and innovation. The findings of the current study provide support for the indirect effect of creativity on entrepreneurship (Fu et al., 2022; Niu and Wu, 2022). The results reinforce the idea that creativity alone will not help in stimulating favorable intentions to start a business. Hence, vocational colleges should change teaching methods and improve students’ understanding and positive attitude toward entrepreneurship. On the other hand, it is also important that vocational colleges help students develop a sense of adventure, reduce the gap between what they perceive as their own subjective risks and the actual risks they face, and enhance their ability to deal with risk in the process of entrepreneurship.

Secondly, entrepreneurs should be provided with a more conducive atmosphere for entrepreneurship by various government departments. On the one hand, with regard to vocational college students, governments need to make the policies more widely known in a timely manner and try to improve the awareness of preferential policies. In addition, the government needs to simplify the application and approval procedures for entrepreneurs. On the other hand, the governments holds various entrepreneurship competitions to build a good stage for college students’ entrepreneurs and create an open entrepreneurial atmosphere to improve students’ intentions to start a business, so as to enhance the possibility of students’ entrepreneurship. In addition to this, they should put forth greater efforts to support business incubators and reevaluate the microfinance programs that are already open to graduates upon completion of their degrees.

### Theoretical implications

In addition, the findings of this study illustrate the meaningful theoretical contributions. First, further expand the scope of creativity research (Wu and Wang, 2018). In the past, the research related to creativity was mostly focused on enterprises, and the research objects were mostly enterprise employees, leaders (Wu and Tian, 2021). This study applies the theory of creativity to the field of vocational education and expands the scope of creativity research by taking students in vocational colleges as the research object. Second, expand the influencing factors of entrepreneurial intention. Although previous studies have also focused on the impact of individual factors on entrepreneurial intention, creativity is still easy to ignore. This study introduces the individual factors of creativity and risk propensity, as well as takes into account environmental factors, such as social support, which enriches the existing literature on incorporating personal ability and environmental factors into intention models. Third, from the perspective of the interaction between individuals and the environment, this paper discusses the potential reasons for the formation of entrepreneurial intention, and clarifies the correlation between multiple variables. Finally, in terms of methodology, this study employs hierarchical modeling using PLS in order to explain the relationships in its model.

## Limitations and directions for future research

Like other studies, this study has certain limitations but provides a certain direction for future research. Firstly, the samples for this study are mainly concentrated in Southeast and East China due to time and energy constraints. Future research can further expand the scope of research and increase the number of samples to improve the validity of research conclusions. Secondly, the gender ratio of the sample in this study is uneven, and the number of females is quite small. Future researchers should pay attention to the sampling balance. Thirdly, this study failed to take into account the discrepancies between an ideal and an actual situation. The true scenario regarding the number of students who actually choose a profession in entrepreneurship after completing their education is unknown. The ways in which our focal individual characteristic (creativity) affects the transition of intentions into actions need additional research (Zampetakis et al., 2011). Finally, in the relationship between creativity and entrepreneurial intention, risk propensity may not only be a moderating variable but also the common influence of other variables, which can also be explored in the future.

## Data availability statement

The raw data supporting the conclusions of this article will be made available by the authors, without undue reservation.

## Author contributions

XW: conceptualization. MW and XN: data curation. XN: writing original draft. XW and ZN: writing—review and editing. All authors contributed to the article and approved the submitted version.

## Funding

This research was supported by the Key Base Project of Science and Technology Project of Education Department of Jiangxi Province in 2018 (no.: GJJ190590), Jiangxi Social Science Planning Project in 2018 (no.: 18JY21), Humanities and Social Science Project of Universities in Jiangxi Province in 2020 (no.: JY20219), Doctoral Research Foundation of Jiangxi Science and Technology Normal University in 2021 (no.:2021BSQD20), Key Research Base of Humanities and Social Sciences in Universities of Jiangxi Province (no. JD18079), National Social Science Foundation of China 2017 Education General Project (no.: BJA170101), and General Project of higher Education and Teaching Reform Research in Hainan Province in 2020: Research on Transformation and Development of Local Undergraduate Colleges in Hainan Province under the Background of Free Trade Zone Construction (no.: HNJG2020-83).

## Conflict of interest

The authors declare that the research was conducted in the absence of any commercial or financial relationships that could be construed as a potential conflict of interest.

## Publisher’s note

All claims expressed in this article are solely those of the authors and do not necessarily represent those of their affiliated organizations, or those of the publisher, the editors and the reviewers. Any product that may be evaluated in this article, or claim that may be made by its manufacturer, is not guaranteed or endorsed by the publisher.
